# Gray matter correlates of attention-deficit hyperactivity disorder in boys versus girls with sensory processing dysfunction

**DOI:** 10.1162/imag_a_00076

**Published:** 2024-02-02

**Authors:** Efstathios D. Gennatas, Jamie Wren-Jarvis, Rachel Powers, Maia C. Lazerwitz, Ioanna Bourla, Lanya T. Cai, Hannah L. Choi, Robyn Chu, Kaitlyn J. Trimarchi, Rafael D. Garcia, Elysa J. Marco, Pratik Mukherjee

**Affiliations:** Department of Epidemiology & Biostatistics, University of California, San Francisco, San Francisco, CA, United States; Department of Radiology & Biomedical Imaging, University of California, San Francisco, San Francisco, CA, United States; Cortica Healthcare, San Rafael, CA, United States; Lifetime Neurodevelopmental Care Center, San Rafael, CA, United States

**Keywords:** ADHD, morphometry, MRI, neurodevelopmental, SPD

## Abstract

Neuroimaging shows volumetric alterations of gray matter in attention-deficit hyperactivity disorder (ADHD); however, results are conflicting. This may be due to heterogeneous phenotypic sampling and limited sensitivity of volumetric analysis. Creating more homogenous cohorts and investigating gray matter microstructure may yield meaningful biomarkers for scientific and clinical applications. Children with sensory processing dysfunction (SPD) have differences in white matter microstructure, but not gray matter volumetric differences. Approximately 40% of SPD children meet research criteria for ADHD. We apply deep learning segmentation of MRI to measure gray matter volume (GMV) and density (GMD) in SPD children with (SPD+ADHD) and without co-morbid ADHD (SPD-ADHD). We hypothesize GMV and GMD are linked to ADHD but with sex-specific regional patterns. We find boys with SPD+ADHD have widespread reduction of GMD in neocortex, limbic cortex, and cerebellum versus boys with SPD-ADHD. The greatest differences are in sensory cortex with less involvement of prefrontal regions associated with attention networks and impulse control. In contrast, changes of ADHD in girls with SPD are in brainstem nuclei responsible for dopaminergic, noradrenergic, and serotonergic neurotransmission. Hence, neural correlates of ADHD in SPD are sexually dimorphic. In boys, ADHD may result from downstream effects of abnormal sensory cortical development.

## 1 Introduction

Attention-Deficit Hyperactivity Disorder (ADHD) is a neurodevelopmental disorder that affects more than 10% of children aged 6-17 years old, with boys twice as likely to be diagnosed as girls and extending into 8% of adults (Bitsko, 2022; Kessler et al., 2005). Attentional challenges, or cognitive control differences, can co-occur with multiple and varied clinical conditions including gross motor, fine motor, language, social, sensory, and emotional challenges. In addition to this constellation of neurodevelopmental variation, ADHD research cohorts often include children with primarily inattentive phenotype, primarily hyperactive/impulsive phenotype, or both (combined); thereby creating significant challenges in obtaining replicable neuroimaging biomarkers. Diagnosis, management, and neuropathological characterization of ADHD remains challenging in part because the neural mechanisms are complex and require more “splitting” rather than “lumping” of affected children to distinguish the relevant differences in neuronal structure. Thus, it is critical to examine potential mediating factors, such as sex, and limit by prevalent clinical conditions, to determine if a more parsimonious approach will yield a replicable imaging biomarker for a more personalized treatment approach. These markers are crucial not only for understanding the condition but also for monitoring change with novel therapeutics, particularly neuromodulation-based approaches which aim to reshape neural connections.

Neuroimaging has been used to establish that children diagnosed with ADHD show gray matter (GM) and white matter (WM) differences from typically developing children (Hoogman et al., 2019); however, findings are largely divergent (Pereira-Sanchez & Castellanos, 2021), in major part due to clinical heterogeneity of the ADHD phenotype (Kuntsi et al., 2014). In their review of 96 pooled neuroimaging studies, including 1914 child and adolescent participants, Samea et al. (2019) report no significant structural or functional differences between the ADHD and non-ADHD cohorts. Interestingly, the sub-analysis did suggest a sex-based difference, with males showing decreased activity in the left inferior frontal gyrus and altered activity in the left pallidum and putamen (subcortical regions). Another functional imaging investigation, aimed at splitting ADHD into more homogeneous “neurocognitive-pathway” cohorts related to executive function/inhibition and/or reward deficits, showed differential activation of brain regions by cohort, suggesting that more specific subtypes might yield more consistent information (Stevens et al., 2018). Thus, studying GM morphometry in a specific population of children at high risk for ADHD may reduce the phenotypic variability inherent to studies using general population ADHD participants and controls and enable identification of brain structural correlates specific to a segment of children affected with ADHD. Children with sensory processing differences present this unique opportunity for creating a more homogenous cohort.

Sensory Processing Dysfunction (SPD), broadly defined, refers to a clinical deficit in the ability to modulate, discriminate, or create an organized response to sensory information, and affects up to 16% of children (Ben-Sasson et al., 2009). Due to the disruptions in sensory processing, children with SPD may demonstrate atypical or delayed intellectual, language, or motor milestones (May-Benson et al., 2009). Of the children with SPD, 40-50% meet research criteria for attention-deficit/hyperactivity disorder (Koziol & Budding, 2012). Hence, the rate of ADHD in SPD is almost five-fold higher than that of the general population of school-age children (American Psychiatric Association, 2013; Faraone et al., 2003). Conversely, children with ADHD are also more likely to have SPD than neurotypical children (Mangeot et al., 2001). This co-occurrence was shown to have functional significance in a study of children with ADHD which found that sensory symptoms accounted for 65% of the variance in academic achievement (Davis et al., 2009). However, the symptoms are not completely overlapping, such that caregiver-reported measures of sensory symptoms can discriminate between ADHD, neurotypical children and other neurodevelopmental disorders such as autism spectrum disorder (ASD) and pervasive developmental disorder (Cheung & Siu, 2009; Dunn & Bennet, 2002). Finally, elevated sensory detection thresholds are strongly associated with hyperactivity (He et al., 2021). While it is not surprising that children who have differences in processing basic sensory information, such as sound, touch, and visual inputs, will also have difficulty in controlling what to attend to and what to ignore, there are limited data exploring the neural basis with this sensory-first approach.

To date, children with SPD have not been reported to have volumetric differences using MR imaging (Chang et al., 2016). However, no prior work has examined detailed GM morphometry in SPD children with ADHD (SPD+ADHD) versus those without (SPD-ADHD). Compared to typically developing peers, school-age children with SPD have reduced WM microstructural integrity on diffusion MRI, especially in posterior tracts that subserve primary and higher-order sensory function as well as cerebellar tracts governing timing of multisensory integration and attention (Chang et al., 2016; Narayan et al., 2021; Owen et al., 2013; Payabvash et al., 2019). A more recent diffusion MRI study has also shown that boys with SPD+ADHD have lower neurite density index, reflecting decreased intracellular volume fraction, throughout projection white matter pathways of the internal capsule and commissural fibers of the splenium of the corpus callosum than boys with SPD but not ADHD (Mark et al., 2023). Furthermore, SPD+ADHD children have reduced midline frontal theta activity on electroencephalography (EEG), a marker of attention abilities captured in real time (Anguera et al., 2017). The midline frontal theta difference is thought to emanate from the dorsal anterior cingulate and adjacent medial prefrontal cortex, limbic and associated neocortical regions that have known importance for impulse control (Ishii et al., 2014). Importantly, after digital brain training for four weeks, the midline frontal theta differences in SPD+ADHD approximated neurotypical peers, providing a model for utilizing a functional biomarker to track brain training with research interventions (Anguera et al., 2017).

While there are overlapping features of ADHD and SPD, they may represent two distinct dimensions that can coexist with unique neurobiological properties (Miller et al., 2012). SPD in the context of ASD and ADHD has been associated with mental health and behavioral challenges, including anxiety, depression, academic difficulties, and disruptive behaviors (MacLennan et al., 2021; Rossow et al., 2021, 2022; Sanz-Cervera et al., 2017). Since SPD as a global cluster of sensory discrimination, modulation, and sensorimotor challenges often but not always co-occurs with attention challenges, it is likely that these information processing functions have both shared and unique aspects of their underlying neural mechanisms. Teasing apart these neural underpinnings will shed light on how these two dimensions thought to be distinct might interrelate (Miller et al., 2012). Indeed, a DTI assessment investigating white matter connectivity underlying attention and visuomotor control in children with SPD showed that there were shared tracts related to performance on related tasks as well as a tract that was uniquely associated with attention, the superior corona radiata (Brandes-Aitken et al., 2018). Additional assessment of cerebellar connections in SPD highlighted the role of the superior and middle cerebellar peduncles in auditory processing, multisensory integration, and attention (Narayan et al., 2021). Historically, the brainstem has been an area of intense investigation for early auditory processing and autism with relatively less investigation for ADHD. However, a study of females with ADHD reported higher values based on auditory brainstem response, specifically in the region from the superior olivary complex to the thalamus (Claesdotter-Hybbinette et al., 2015). Investigating the structural gray matter differences for all brain regions, including the information processing networks of the brainstem, subcortical gray matter, neocortex, limbic cortex, and cerebellum within the context of sex differences, is a critical next step (Gershon, 2002; Osorio et al., 2021).

T1-weighted (T1w) MRI is the most common sequence collected in both clinical and research settings. It offers high-resolution structural images of the brain, with high tissue contrast, allowing for accurate segmentation of GM, WM, and cerebrospinal fluid (CSF). Multiple metrics can be derived from a single T1-weighted imaging volume, such as GM volume (GMV), density (GMD), and their multiplicative product, mass (GMM). These morphological metrics may offer complementary information on brain structure and pathology. Studying GM morphometry in a specific population of children at high risk for ADHD may reduce the phenotypic variability of prior ADHD neuroimaging studies using general population control participants and thereby enable better sensitivity for identifying the brain structural correlates of ADHD.

One hypothesis for the high rate of ADHD in SPD is that abnormal early development of sensory pathways results in downstream effects on neocortical attentional circuits, limbic emotional regulatory circuits, and cerebellocortical timing circuits that require precisely coordinated sensory feedback for normal maturation. A corollary would be that more aberrant sensory GM development would raise the risk for ADHD. Therefore, we postulate reductions of neocortical, limbic, and cerebellar regional GMV, reflecting macrostructure, and GMD, reflecting microstructure, in SPD+ADHD compared to SPD-ADHD. To test this hypothesis, we use a custom T1-preprocessing pipeline to perform state-of-the-art brain tissue segmentation, parcellation, and extraction of GMD and GMV to compare children with SPD who satisfy criteria for ADHD with those with SPD who do not. We separately analyze boys and girls for evidence of sexual dimorphism, given the different clinical phenotypes of ADHD in males versus females (Gershon, 2002; Martin et al., 2018; Mowlem et al., 2019; Murray et al., 2019). Supportive evidence for this hypothesis would establish important brain structural correlates of ADHD that could also be tested in non-SPD populations. This would also help pave the way towards developing clinically useful neuroimaging biomarkers of ADHD for clinical research trials and eventually routine clinical practice, which remains a major unmet need.

## 2 Materials and Methods

### 2.1 Participants

We prospectively enrolled children between 8-12 years of age at a community neurodevelopmental clinic (NDC). The research protocol was approved by the institutional review board at our medical center with written informed consent obtained from the parents or legal guardians and assent obtained from the study participants. Exclusion from the study is based on the following criteria:

- Nonverbal Index ≤ 70 on the Wechsler Intelligence Scale for Children, Fifth Edition
- < 1 “Yes” or < 2 “Maybe / A Little” responses on the ESSENCE-Q-REV parent questionnaire for neurodevelopmental concerns
- Caregiver(s) unable to complete intake forms
- In utero toxin exposure
- Gestational age < 32 weeks or intrauterine growth restriction (birth weight < 1500 grams)
- Hearing or visual impairment
- Additional medical/neurologic condition, including active epilepsy, malignancy, or known brain injury/malformation
- Research designation of ASD based on the Social Communications Questionnaire and the Autism Diagnostic Observation Scale, 2^nd^ edition

All participants were assessed for SPD using the Short Sensory Profile (Licciardi & Brown, 2021; McIntosh et al., 1999) caregiver questionnaire. A score of ≥2 standard deviations from the mean in any of the following domains corresponds to an SPD designation: tactile sensitivity, taste/smell sensitivity, movement sensitivity, under-responsive/seeks sensation, auditory filtering, low energy/weak, and visual/auditory sensitivity. ADHD was assessed with the Behavior Assessment System for Children: Third Edition (BASC-3) with a categorization of clinical significance, using a 95th percentile threshold, corresponding to an ADHD label (Reynolds & Kamphaus, 2015).

### 2.2 Neuroimaging assessment

All subjects were imaged on a single Siemens 3 Tesla (3 T) Prisma MRI scanner (Erlangen, Germany) using a 64-channel head coil. T1-weighted imaging of the whole brain was acquired with an axial 3D magnetization prepared rapid acquisition gradient-echo (MPRAGE) T1-weighted sequence with 1 mm voxel resolution along all three spatial dimensions (TE = 2.9 ms, TR = 2300 ms, TI = 900 ms). T1w scans with unacceptable levels of motion artifact on visual inspection by a pediatric neuroradiologist (P.M.) were excluded from analysis.

### 2.3 MR image preprocessing

**Fig. 1.**
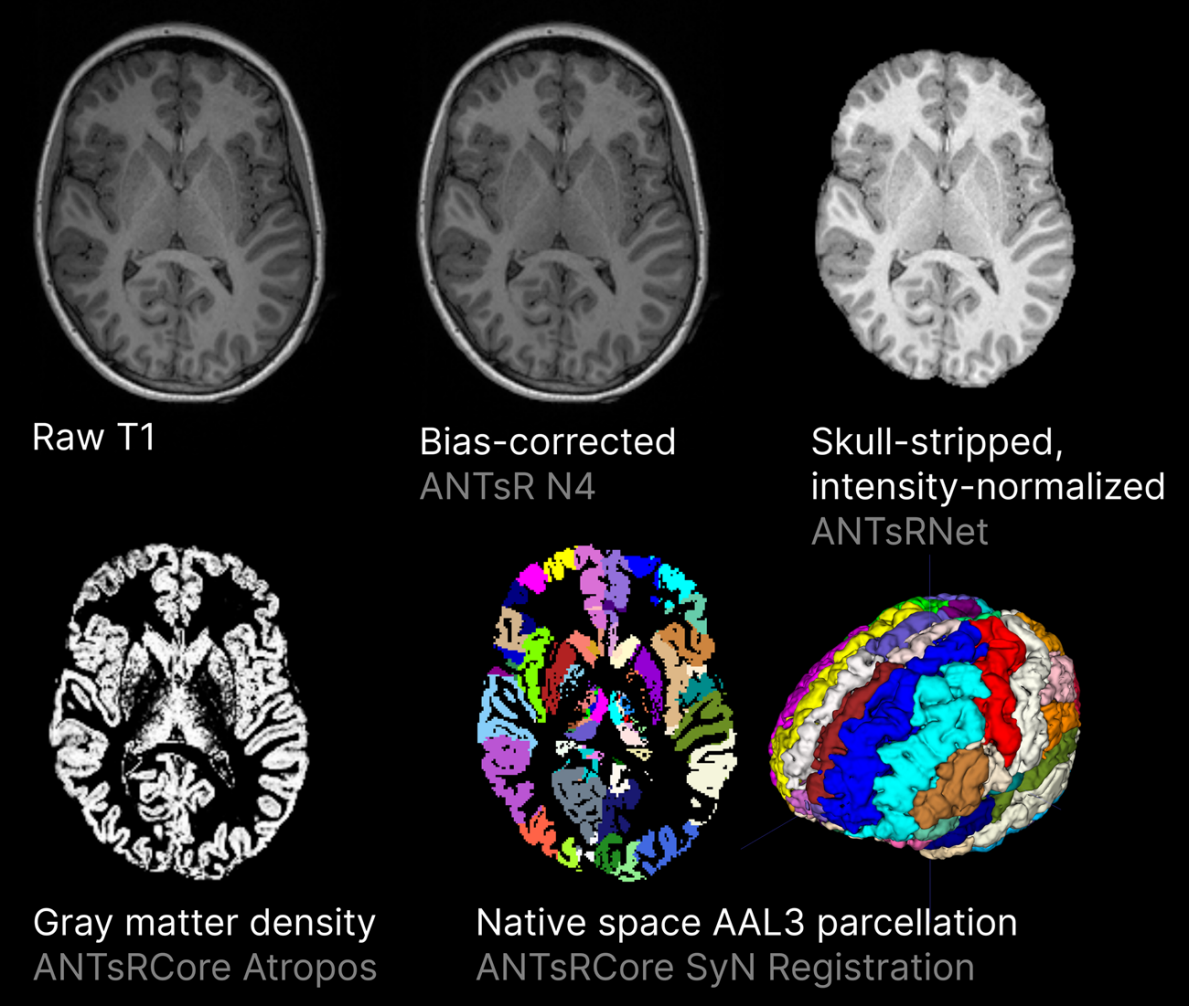
3D T1-weighted Image Volume Preprocessing.

T1-weighted images were preprocessed using a custom pipeline based on previous analysis of a large number of children’s brain images (Gennatas et al., 2019), as illustrated in Figure 1. The new pipeline is based on ANTsR and ANTsRNet (Tustison et al., 2019, 2021) and is made available in the prprcss R package (https://github.com/egenn/prprcss). Raw T1 volume were bias-corrected using the N4 algorithm (Tustison et al., 2010). Brain extraction was performed using ANTsRNet’s brainExtraction tool, which uses a pretrained 3D U-net. Bias-field corrected brain-extracted volumes were registered to brain-only template in MNI space using SyN Symmetric Diffeomorphic registration (Avants et al., 2008). Three-class tissue segmentation was performed using Atropos (Avants et al., 2011) using unbiased 3-class K-means initialization. The Automated Anatomical Labelling Atlas version 3 (AALv3) as reported by Rolls et al. (2020) was transformed into each subject’s native space by applying the inverse of the native-to-template space transformation. Regional GMD and GMV were extracted for each AALv3 region using the *labelstat_native* function. GMD was defined as the mean gray matter tissue probability output by Atropos, and volume was estimated as the physical native space volume of each region. In both cases, only voxels classified as gray matter were included.

### 2.4 Statistical analysis

All statistical analysis and visualization was performed using the rtemis package (Gennatas et al., 2019) running in R version 4.2.1 (R Core Team, 2022). Boxplots were drawn to show distribution of GMD and GMV in SPD+ADHD versus SPD-ADHD. Linear models were fit to regress each region’s GMD and GMV on ADHD status, while correcting for age, sex, and full-scale IQ from the Wechsler Intelligence Scale for Children Fifth Edition (WISCV), using the *massGLM* function.

For each AALv3 region *i*:

$${\left\{GMD,GMV\right\}}_{i}\sim ADHD+Age+Sex+IQ$$

To present whole-brain mass-univariate results, “volcano” plots were created to visualize p-values against linear coefficients in all AALv3 gray matter regions.

### 2.5 Classification

Classification models were trained to predict SPD+ADHD versus SPD-ADHD status. Given the small sample size, instead of training models using multiple algorithms and performing comprehensive hyperparameter tuning, we used a single algorithm, LightGBM, a highly efficient and flexible gradient boosting implementation (Ke et al., 2017). Hyperparameters were fixed using conservative values to provide increased regularization and avoid overfitting (max number of leaves = 4, learning rate = 0.001, lambda L1 = 1, lambda L2 = 1, max iterations = 1000 with early stopping). Two sets of classification models were trained, one using GMD and the other using GMV data along with age and sex in both cases.

## 3 Results

### 3.1 Demographics

Of the 136 children screened with NDC, 79 children (57 male, 22 female) met inclusion and exclusion criteria for the study and had T1w scans of acceptable quality (Table 1). This SPD cohort was further split by ADHD as defined by the BASC. Thirty-four children (43%) met criteria for SPD and ADHD (SPD+ADHD). Forty-five children did not exceed the ADHD threshold and thus were placed in the SPD-only cohort (SPD-ADHD). Fisher’s exact test suggests no association of sex and ADHD status.

**Table 1.** Participant characteristics.

	Overall(N = 79)	SPD-ADHD(N = 45)	SPD+ADHD(N = 34)
Age			
Mean (SD)	10.1 (1.60)	10.3 (1.62)	9.79 (1.55)
Median [Min, Max]	9.78 [8.03, 13.0]	10.4 [8.03, 13.0]	9.46 [8.03, 13.0]
Sex			
Male	57 (72.2%)	32 (71.1%)	25 (73.5%)
Female	22 (27.8%)	13 (28.9%)	9 (26.5%)

### 3.2 Gray matter analysis

**Fig. 2.**
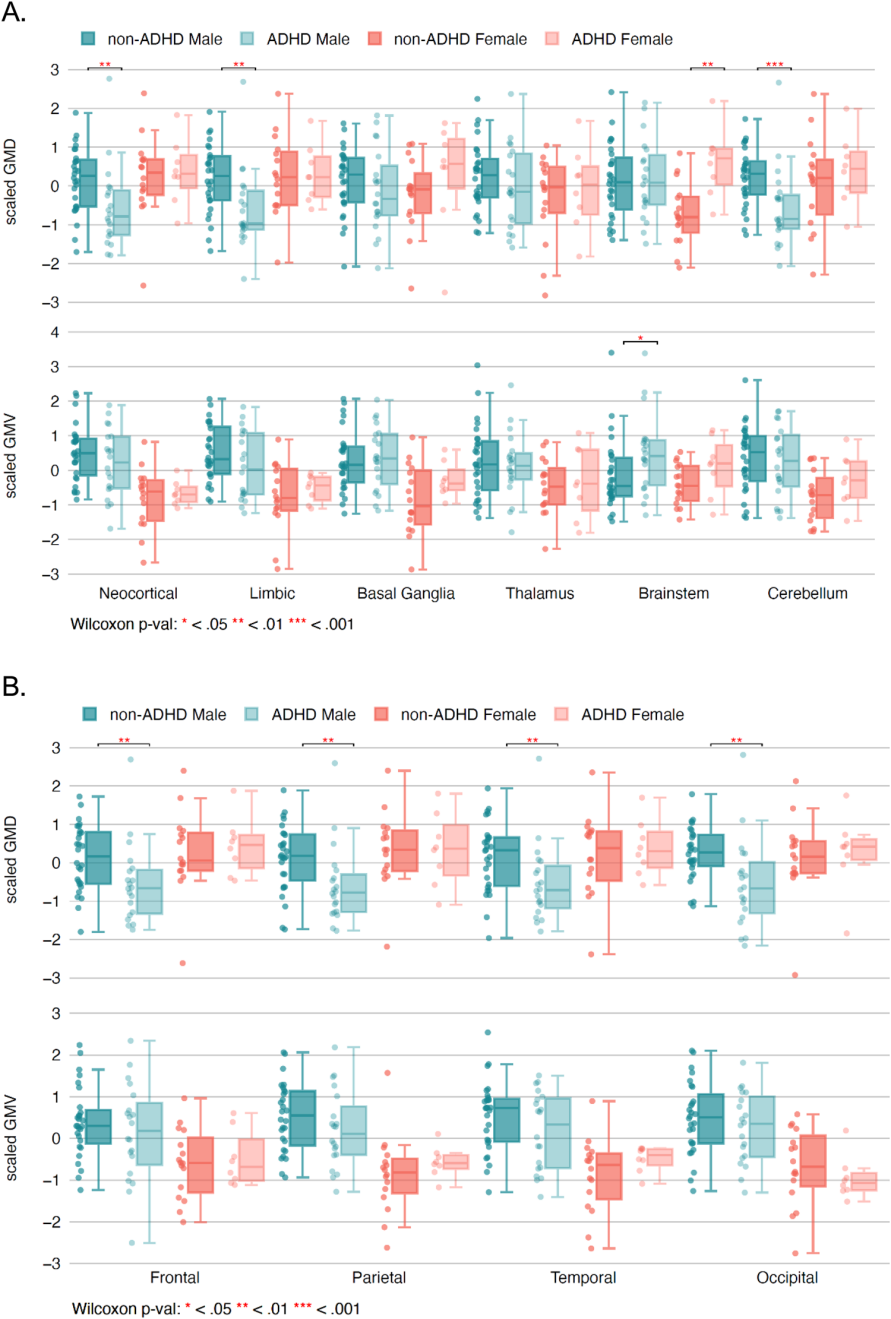
Gray Matter Density (GMD) and Volume (GMV) in ADHD versus non-ADHD. Scaled GMD and GMV are presented to normalize for the large inter-regional variation in density and volume, for example, between neocortex and brainstem.

Boxplots were drawn to show mean GMD and GMV (Fig. 2A) by general brain region (brainstem, basal ganglia, thalamus, limbic cortex, neocortex, cerebellum), stratified by ADHD status and Sex. Neocortex was also further subgrouped by cerebral lobes (frontal, parietal, temporal, occipital) in Figure 2B.

In males, group mean GMD was significantly lower for SPD+ADHD versus SPD-ADHD in the neocortex (Cohen’s *d* = -0.79), limbic system (*d* = -0.83), and cerebellum (*d* = -0.88), but did not differ in basal ganglia, thalamus, or brainstem. For neocortex in males, this lower GMD was found in all four cerebral lobes: frontal (*d* = -0.72), parietal (*d* = -0.77), occipital (*d* = -0.86), and temporal (*d* = -0.73). In contrast, for females, group mean GMD was significantly higher for SPD+ADHD versus SPD-ADHD in the brainstem (*d* = 1.61), but did not differ in neocortex, limbic system, basal ganglia, thalamus, or cerebellum.

Unlike GMD, but similar to previous reports, group mean GMV did not show pronounced differences between SPD+ADHD and SPD-ADHD individuals for males or females, aside from higher GMV in males for the brainstem.

#### 3.2.1 Atlas-based regional gray matter density and volume

**Fig. 3.**
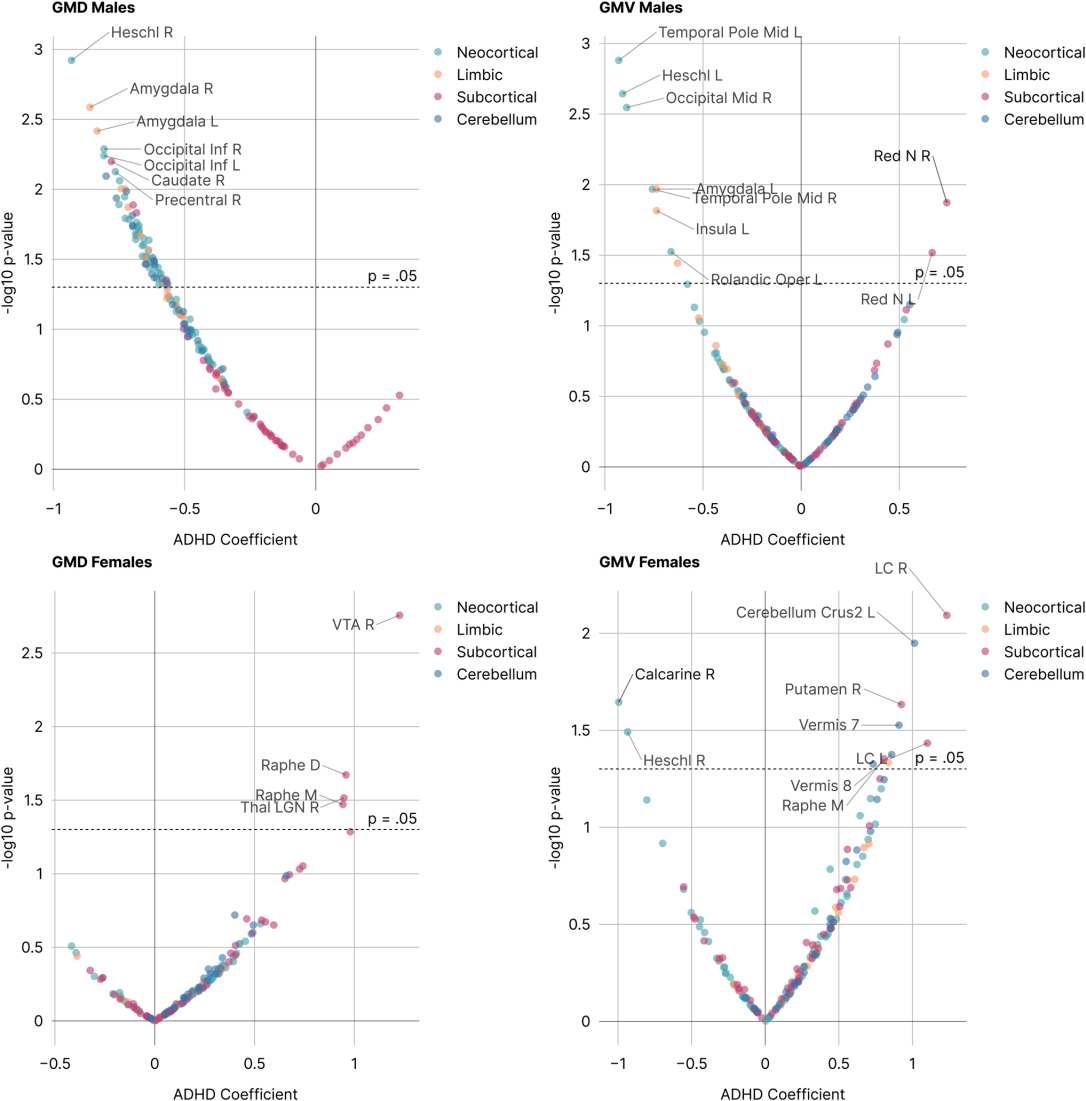
Volcano Plots of GMD and GMV in ADHD versus non-ADHD in AALv3 Brain Atlas Regions. Increasingly negative numbers on the x-axis indicate decreasing density or volume, whereas increasingly positive numbers on the x-axis indicate increasing density or volume. The y-axis denotes the statistical significance level with p = 0.05 (uncorrected for multiple comparisons) indicated by the dashed line. AALv3 region name abbreviations are provided in Rolls et al., 2020.

For exploratory region-specific analysis, volcano plots were generated to show ADHD coefficients for all AALv3 brain parcels against the negative log base 10 of the p-value (Fig. 3), corrected for age and full-scale IQ. Table 2 summarizes these mass-GLM results. GMD and/or GMV were *negatively* correlated with ADHD in boys, especially sensorimotor regions such as auditory cortex (Heschl’s gyrus, temporal pole), visual cortex (calcarine gyrus, inferior and middle occipital gyri, and lingual gyrus), olfactory cortex, and primary motor cortex (precentral gyrus), as well as limbic regions such as the amygdala, insula, and hippocampus. For girls, there were fewer regional associations with ADHD and most of these were *positive* correlations of subcortical nuclei GMV. Examples include the major brainstem centers for neurotransmitter production such as the locus coeruleus (norepinephrine), ventral tegmental area (dopamine), and the dorsal and median raphe nuclei of the midbrain (serotonin). Other regions positively correlated with ADHD in girls were found in the cerebellum (vermis and crura) and the thalamus, especially the lateral geniculate nucleus which is responsible for visual neurotransmission to primary visual cortex. Like boys, there were also negative correlations of Heschl’s gyrus (primary auditory cortex) and calcarine gyrus (primary visual cortex) with ADHD in girls.

**Table 2.** Gray matter regional correlation with ADHD in SPD children.

Males						Females					
GMD			GMV			GMD			GMV		
Region	r	p-value	Region	r	p-value	Region	r	p-value	Region	r	p-value
Heschl_R	-0.931	**0.0011**	Occipital_Mid_R	-0.889	**0.0028**	Vermis_7	0.659	0.1033	Calcarine_R	-0.994	**0.0226**
Amygdala_R	-0.861	**0.0025**	Amygdala_L	-0.736	**0.0107**	Cerebellum_10_L	0.401	0.1907	Vermis_7	0.908	**0.0297**
Amygdala_L	-0.834	**0.0038**	PHG_L	-0.630	**0.0358**	CRcr-I_L	0.484	0.2565	Heschl_R	-0.935	**0.0322**
Occipital_Inf_R	-0.807	**0.0051**	Cerebellum_10_L	0.553	0.0707	OFCpost_L	-0.417	0.3099	Vermis_8	0.859	**0.0421**
Occipital_Inf_L	-0.808	**0.0057**	Temporal_Inf_L	-0.544	0.0740	Rectus_L	-0.394	0.3440	Amygdala_R	0.836	**0.0463**
Caudate_R	-0.779	**0.0063**	Hippocampus_L	-0.522	0.0879	Amygdala_R	-0.389	0.3628	CRcr-II_R	0.733	**0.0471**
Precentral_R	-0.764	**0.0074**	PCL_R	0.525	0.0903	Cerebellum_8_L	0.339	0.3721	Parietal_Sup_L	0.788	0.0632
Cerebellum_9_R	-0.799	**0.0080**	Postcentral_R	-0.493	0.1111	Precentral_L	0.338	0.4191	Calcarine_L	-0.804	0.0722
Fusiform_L	-0.748	**0.0086**	Vermis_10	0.488	0.1153	CRcr-I_R	0.330	0.4380	IFGtr_R	0.645	0.0870
Hippocampus_R	-0.741	**0.0098**	Parietal_Sup_R	-0.441	0.1573	Frontal_Mid_2_R	0.318	0.4414	Fusiform_L	0.699	0.1154
PHG_L	-0.727	**0.0099**	TPsup_R	-0.414	0.1822	Cerebellum_9_R	0.301	0.4458	Cuneus_L	-0.696	0.1210
Cerebellum_10_L	-0.722	**0.0103**	ACC_sub_R	-0.398	0.1884	Vermis_8	0.305	0.4777	Hippocampus_L	0.704	0.1213
OFCpost_L	-0.729	**0.0112**	Precuneus_L	-0.393	0.2012	Cerebellum_9_L	0.278	0.4784	Fusiform_R	0.662	0.1410
Vermis_8	-0.760	**0.0115**	Parietal_Sup_L	-0.394	0.2043	Vermis_10	0.321	0.4784	Cerebellum_8_L	0.550	0.1498
Lingual_R	-0.750	**0.0128**	CR-IV_L	0.376	0.2282	Occipital_Inf_L	0.308	0.4794	TPsup_L	0.623	0.1553
N_Acc_R	-0.696	**0.0129**	Hippocampus_R	-0.359	0.2514	Parietal_Sup_R	-0.303	0.4967	PHG_L	0.608	0.1850
PHG_R	-0.715	**0.0134**	Heschl_R	-0.349	0.2591	N_Acc_R	-0.260	0.5084	N_Acc_L	0.580	0.2044
Caudate_L	-0.683	**0.0147**	Cerebellum_9_R	0.339	0.2718	OFCpost_R	-0.262	0.5096	Rectus_L	-0.555	0.2086
Vermis_10	-0.699	**0.0153**	Occipital_Sup_L	-0.324	0.2875	Occipital_Sup_L	0.288	0.5138	Lingual_R	0.555	0.2190
Calcarine_R	-0.726	**0.0161**	Occipital_Sup_R	-0.314	0.2937	CRcr-II_R	0.266	0.5314	Amygdala_L	0.479	0.2585
Olfactory_R	-0.712	**0.0164**	Cerebellum_9_L	0.317	0.3094	Temporal_Inf_L	0.238	0.5772	PHG_R	0.497	0.2742
Precuneus_L	-0.683	**0.0172**	CRcr-I_R	-0.292	0.3112	OFGinf-II_R	0.229	0.5878	CR-IV_L	0.442	0.2948
Vermis_7	-0.698	**0.0181**	Cingulate_Post_R	-0.312	0.3160	IFGtr_R	0.234	0.5899	Temporal_Inf_L	0.484	0.2966
PCL _R	-0.674	**0.0181**	Cerebellum_3_L	0.304	0.3279	Occipital_Inf_R	0.243	0.6003	TPsup_R	0.450	0.2974
CRcr-I_R	-0.698	**0.0184**	Amygdala_R	-0.277	0.3583	Lingual_R	0.216	0.6287	OFCmed_L	-0.446	0.3251

BOLD: p-values less than 0.05 (uncorrected). AALv3 region name abbreviations are provided in Rolls et al., 2020.

**Fig. 4.**
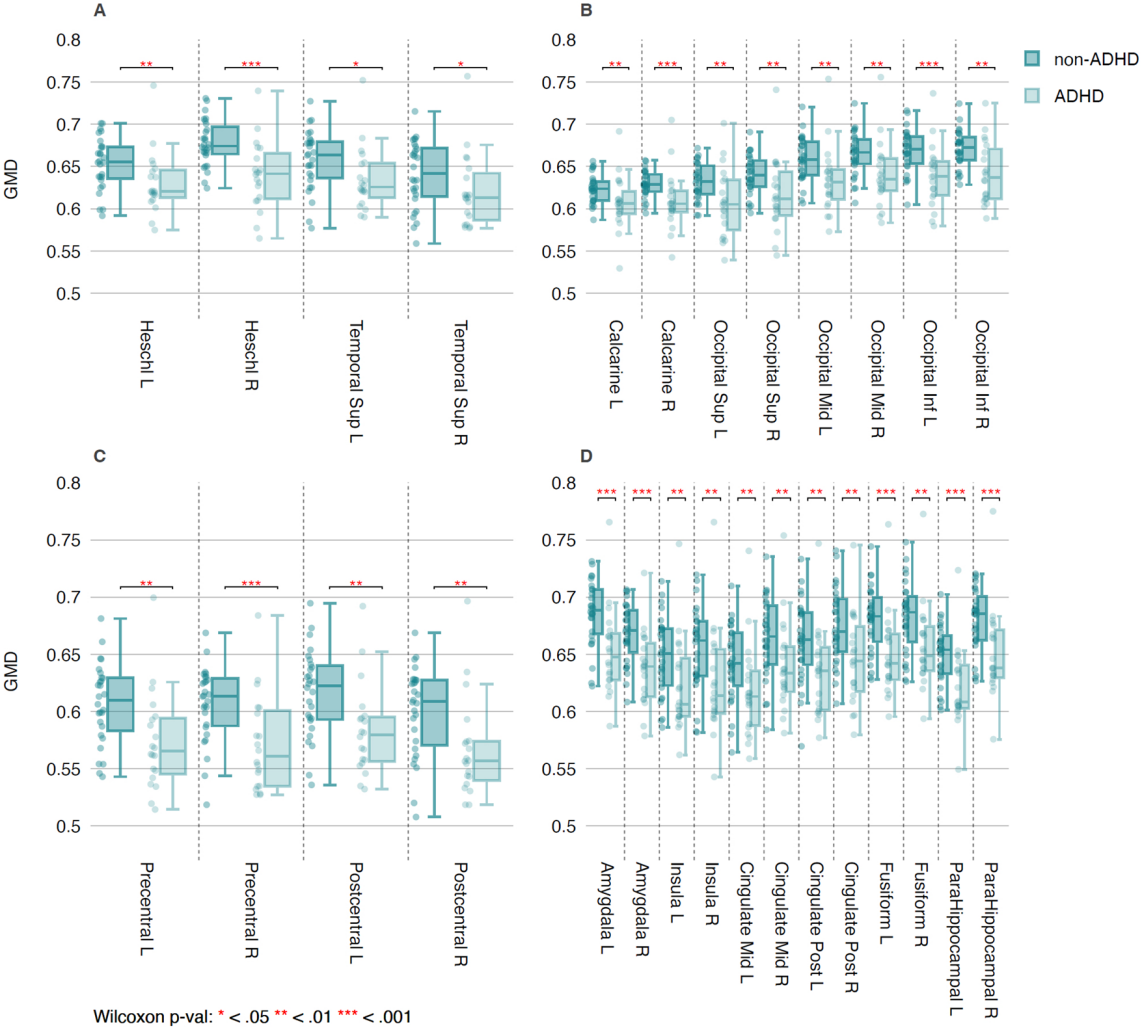
GMD in Males for (A) Auditory, (B) Visual, (C) Somatomotor, and (D) Limbic Cortex. AALv3 region name abbreviations are provided in Rolls et al., 2020.

Boxplots of GMD in males for selected AALv3 parcels involved in sensorimotor or limbic function are shown in Figure 4. Exploratory analysis, uncorrected for multiple comparisons, shows significantly lower GMD in boys with SPD+ADHD than those with SPD-ADHD in all examined regions, including primary auditory cortex (Heschl’s gyrus), higher-order auditory cortex (superior temporal gyrus), primary visual cortex (calcarine gyrus), higher-order visual cortex (superior, middle, and inferior occipital gyri), primary somatosensory cortex (postcentral gyrus), and primary motor cortex (precentral gyrus). Lower GMD in boys with SPD+ADHD than those with SPD-ADHD were also found throughout limbic cortex, including the amygdala, mid- and posterior cingulum, ventral cingulum (parahippocampal gyrus), fusiform gyrus, and insula.

### 3.3 Classification results

**Fig. 5.**
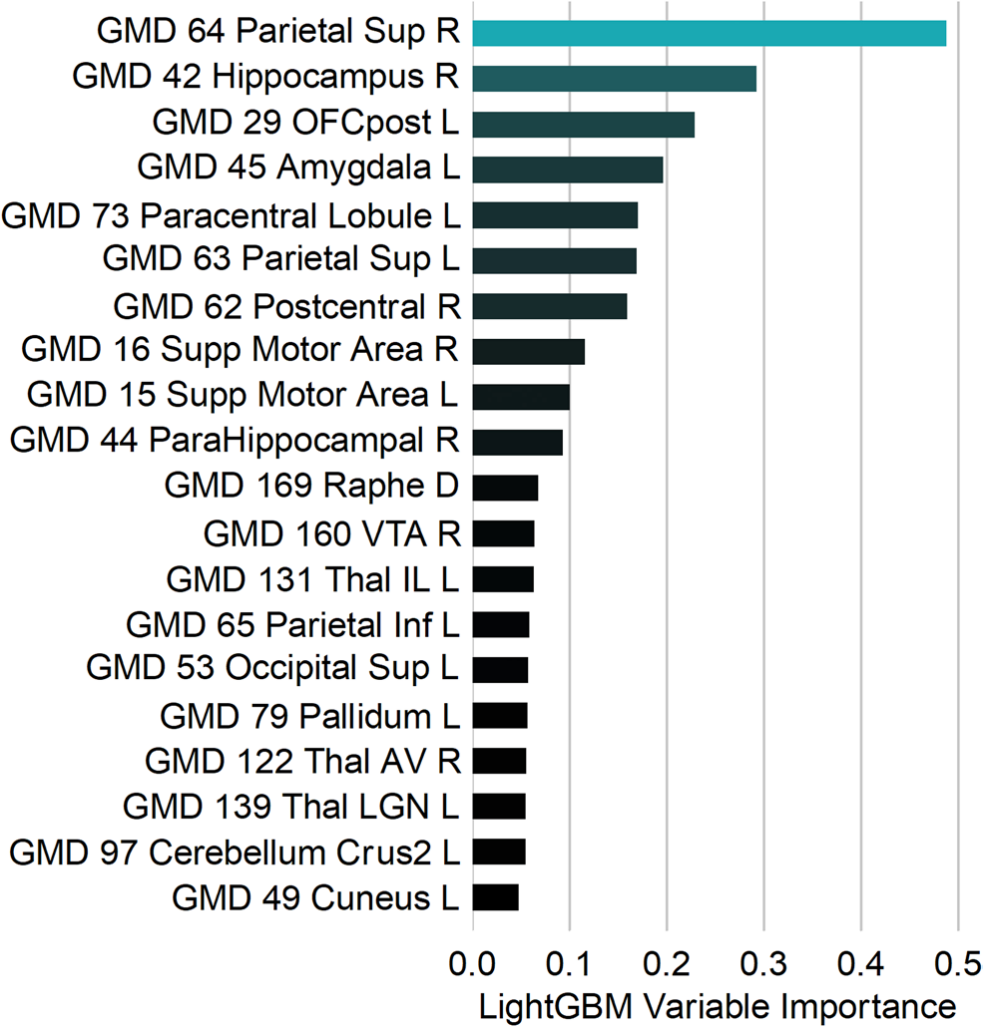
Top 20 AALv3 Brain Regions by Importance in the GMD Classification Model for Distinguishing SPD+ADHD from SPD-ADHD. AALv3 region name abbreviations are provided in Rolls et al., 2020.

LightGBM classification models trained using GMD data achieved a mean area under the curve (AUC) across 25 stratified subsamples of 0.74 (sd = 0.15), compared to 0.57 (sd = 0.18) for models trained using GMV. Consistent with the mass-univariate results, this suggests that GMD is better able to capture regional brain patterns that distinguish SPD+ADHD from SPD-ADHD subjects. Figure 5 shows the relative variable importance of the top 20 features for the GMD model. Variable importance is a unitless, directionless metric that estimates the total contribution of each feature in the prediction of the outcome of interest, which includes potential interactions and non-linearities.

## 4 Discussion

### 4.1 Neuroimaging of ADHD

ADHD remains a diagnostic and management challenge affecting a large proportion of children, in part due to the failure to dissect the neural underpinnings of the condition. Like many neuropsychiatric disorders, extensive previous work suggests clinical heterogeneity (Kuntsi et al., 2014). As a result, definitive neuroimaging signatures of ADHD have proven elusive (Pereira-Sanchez & Castellanos, 2021). A recent mega-analysis of over 2000 ADHD children aged 4-14 years versus typically developing controls has shown GM morphometric differences with small effect sizes that are largely limited to the frontal lobes and, to a lesser extent, the temporal lobes (Hoogman et al., 2019). An even more recent analysis of over 7800 school-age children participating in the Adolescent Brain Cognitive Development (ABCD) study also found reduced GM volume and surface area changes of ADHD that were greatest in the frontal and temporal lobes (Lin et al., 2023). In this work, we focus on a more homogenous sample of school-age children with sensory processing challenges, a condition with high ADHD comorbidity, and utilize cutting-edge structural MR imaging morphometric analysis to identify gray matter correlates of attention-deficit hyperactivity disorder in this population. This work is novel in that GM morphometry has not yet, to our knowledge, yielded brain volumetric features associated with SPD. The high quality of the GMD data is illustrated by the ability to discriminate known neurobiological features in both the SPD+ADHD and SPD-ADHD groups, such as the relatively low GMD of the calcarine cortex compared to higher-order visual cortex of the middle and inferior occipital gyri, due to the presence of the heavily myelinated stria of Gennari at layer IVb of primary visual cortex.

### 4.2 Sex differences in the gray matter density correlates of ADHD in children with SPD

We observe *reduced* GMD in boys with SPD+ADHD versus SPD-ADHD, especially in neocortical, limbic, and cerebellar regions. Interestingly, girls show the converse GM group differences, with *increased* GMD in SPD+ADHD compared to SPD-ADHD in the brainstem. This suggests a strong sexual dimorphism in the underlying neural basis for ADHD in the school-age SPD population, which perhaps reflects differences in the clinical phenotypes of boys versus girls, where boys show more hyperactivity and impulsiveness whereas girls display more inattentiveness (Gershon, 2002; Martin et al., 2018; Mowlem et al., 2019; Murray et al., 2019). GMV did not show as pronounced an effect of ADHD as did GMD. This indicates that the GM differences are more at the microstructural level involving the myeloarchitectonics probed by GMD T1-weighted tissue segmentation than at the macrostructural level measured by volumetrics, although significant effects can be found when analyzing thousands of participants (Hoogman et al., 2019; Lin et al., 2023). This highlights the innovative approach to GM morphometry employed in our investigation (Gennatas et al., 2019). Indeed, the effect sizes of the differences in neocortical GMD between boys with SPD+ADHD and those with SPD-ADHD in our study, which was greatest in the occipital lobes (*d* = -0.86) and least in the frontal lobes (*d* = -0.72), were all much larger than the traditional volumetric measures such as GMV and cortical thickness and surface area employed in Hoogman et al. (2019) which ranged from *d* = -0.10 to at most *d* = -0.21. Sexually dimorphic microstructural differences between school-age SPD+ADHD and SPD-ADHD cohorts are also observed in white matter using diffusion MRI, again with larger effects in boys than girls (Mark et al., 2023).

Regional GMD metrics enabled 74% accuracy (AUC = 0.74) in distinguishing SPD+ADHD from SPD-ADHD across both boys and girls in an exploratory classification analysis, whereas regional GMV performed at 57% accuracy (AUC = 0.57) which is at chance levels. The regions for which GMD best determined ADHD included limbic-related regions such as hippocampus, amygdale, and orbitofrontal cortex (OFC) as well as bilateral superior parietal regions known to be involved in attention networks. This corresponds well with the known attentional and impulsivity phenotypes of ADHD, but these promising exploratory results need to be confirmed and extended in a larger cohort.

### 4.3 Neocortical, limbic, and cerebellar GMD correlates of ADHD in boys with SPD

The decreased GMD of sensory regions in males with SPD+ADHD versus SPD-ADHD, with concomitantly reduced GMD in limbic and cerebellar regions, provides initial support for the hypothesis that abnormal sensory gray matter microstructure during brain development may lead to downstream effects on the maturation of attentional and emotional regulation pathways as well as cerebellocortical timing circuits. We have previously shown white matter microstructural deficits in cerebral sensory tracts and corticocerebellar tracts using diffusion MRI in school-age children with SPD compared to typically developing controls (Chang et al., 2016; Narayan et al., 2021; Owen et al., 2013; Payabvash et al., 2019). The observation of reduced GMD of the primary motor cortex in the precentral gyrus in males with SPD+ADHD compared to those with SPD-ADHD is not surprising since school-age boys, but not girls, with ADHD display fine motor control deficits (Cole et al., 2008; Hasson & Fine, 2012). Notably, prefrontal regions classically associated with attention networks are *not* represented in the AALv3 GM regions most correlated with ADHD status in our findings, suggesting that greater structural gray matter abnormalities of sensory, limbic, and cerebellar circuits are most involved in the development of ADHD in this population. This is concordant with recent findings from dynamic resting-state functional MRI (fMRI) that finds sex differences in ADHD, including differences in the interactions of sensory networks such as the visual network, and of cerebellar networks, with the task-negative default mode network (Agoalikum et al., 2023). However, given the modest sample size of this exploratory analysis of AALv3 regions, we cannot exclude small effect sizes for prefrontal attention centers in ADHD status for the SPD population; therefore, further hypothesis-driven investigation in larger cohorts is needed.

### 4.4 Brainstem gray matter correlates of ADHD in girls with SPD

However, not as much evidence for the sensory hypothesis is apparent in females, with only right calcarine gyrus (primary visual cortex) and right Heschl’s gyrus (primary auditory cortex) showing significant reductions of GMV in SPD+ADHD versus SPD-ADHD in exploratory analysis. Therefore, it is possible that a different neural mechanism may predominate in girls. One possibility raised by our results is that ADHD in girls with SPD may be mediated by the brainstem, which has increased GMD in those with SPD+ADHD with a particularly large effect size (*d* = 1.61). Exploratory analysis of AALv3 brainstem regions shows that many of the most affected areas in girls with SPD+ADHD are those associated with neurotransmitter regulation of cerebral function, including the ventral tegmental area for dopamine, the locus coeruleus for norepinephrine, and the dorsal and median raphe nuclei for serotonin. This suggests potential therapeutic targets for girls with ADHD, since a variety of FDA-approved medications exist for modulating dopaminergic, serotonergic, and noradrenergic neurotransmission. However, one limitation of this pilot study of ADHD in SPD is the small sample size of girls, who have a considerably lower risk of both SPD and ADHD than boys. Larger multicenter studies are needed for more definitive conclusions about the association between SPD and ADHD in females. Another shortcoming of this initial investigation is the lack of long-term follow-up in these participants, which will be forthcoming in future longitudinal studies.

### 4.5 Limitations and future directions

Our sample size did not allow a comprehensive supervised learning analysis, which would include training models using multiple machine-learning algorithms and extensive hyperparameter tuning. As more cases and features (clinical, imaging, genomic, etc.) become available, such models will be able to provide more insights into both pathophysiology and heterogeneity as well as contribute to the diagnosis and monitoring of individuals.

More investigation is needed in children with ADHD who do not exhibit SPD to test whether the neuroimaging features uncovered in our study generalize to this more heterogeneous population. Further research is also needed at earlier stages of development to explore the link between sensory function and executive function. Multimodal investigations incorporating noninvasive electrophysiology such as high-density EEG and MEG might also elucidate this important question by leveraging high temporal resolution mapping of cortical function. The discovery of objective quantitative brain imaging biomarkers of SPD and related ADHD comorbidities, which are currently entirely lacking, would greatly accelerate clinical research into new treatment strategies, ultimately mitigating the weighty personal, familial, and societal burdens from these very prevalent neurodevelopmental disorders.

## Data Availability

All data used for this report are available at the NIH Data Archive (https://nda.nih.gov) Accession Number: 4095004. The code used for data analysis is available upon request.
